# Specific and rapid identification of the *Pheretima aspergillum* by loop-mediated isothermal amplification

**DOI:** 10.1042/BSR20181943

**Published:** 2019-02-22

**Authors:** Qing Huang, Zhiwu Li, Zhiguo Ma, He Li, Runqian Mao

**Affiliations:** 1 School of Basic Courses, Guangdong Pharmaceutial University, Guangzhou 510006, China; 2 Guangdong Key Laboratory of Animal Conservation and Resource Utilization, Guangdong Public Laboratory of Wild Animal Conservation and Utilization, Guangdong Institute of Applied Biological Resources, Guangzhou 510260, China; 3 Research and Development Department, Breeding Base of Guang-dilong, Guangxi 537700, China; 4 College of Pharmacy, Jinan University, Guangzhou 510632, China

**Keywords:** loop-mediated isothermal amplification (LAMP), mitochondrial cytochrome C oxidase I (COI), Pheretima aspergillum, rapid identification

## Abstract

Guang-dilong (*Pheretima aspergillum*) is a traditional Chinese animal medicine that has been used for thousands of years in China. In the present study, we purposed to establish a new rapid identification method for Guang-dilong. We provided a useful technique, loop-mediated isothermal amplification (LAMP), to differentiate Guang-dilong from other species. Four specific LAMP primers were designed based on mitochondrial cytochrome *c* oxidase I (COI) gene sequences of Guang-dilong. LAMP reaction, containing DNA template, four primers, 10× Bst DNA polymerase reaction buffer, dNTPs, MgSO_4_, and Bst DNA polymerase, was completed within 60 min at 63°C. The LAMP product can be visualized by adding SYBR Green I or detected by 2% gel electrophoresis. LAMP technology was successfully established for rapid identification of Guang-dilong. In addition, DNA template concentration of 675 fg/μl was the detection limit of LAMP in Guang-dilong, which was 1000-times higher than conventional PCR. The simple, sensitive, and convenient LAMP technique is really suited for on-site identification of Guang-dilong in herbal markets.

## Introduction

**Figure 1:**
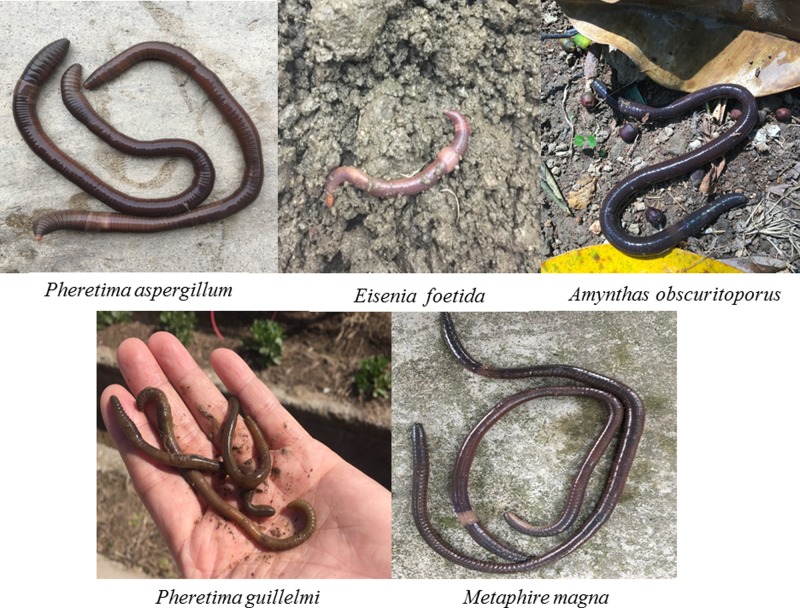
Five different species of earthworm *Pheretima aspergillum, Eisenia foetida, Amynthas obscuritoporus, Pheretima guillelmi*, and *Metaphire magna*.

Historically and traditionally, earthworms are widely used as medicine for treatment of various diseases with its many medicinal effects in Asia [1–7]. Guang-dilong (*Pheretima aspergillum*), commonly known as the best quality, is one of species of earthworms that is recorded by the Chinese Pharmacopoeia. Although these species (*Pheretima aspergillum, Eisenia foetida, Amynthas obscuritoporus, Pheretima guillelmi*, and *Metaphire magna*) have similar appearance (Figure 1), they do not have the same medicinal value as Guang-dilong, which is the main problem effecting the quality of herbal medicine. In recent years, with the price of commercial Guang-dilong growing rapidly, many different species and a wide range of sources sprang up in herbal markets, thus it increases the difficulty for consumers to quickly differentiate amongst them. The traditional morphological identification has been unable to accurately determine species of earthworms due to their similar morphological features. In particular, when the Guang-dilong was processed into dried medicinal herbs, the difficulty in distinguishing their species is increased, so the adulterants of Guang-dilong were commonly found in the herbal markets. Compared with traditional morphological and chemical detection, molecular techniques are more accurate and have long been applied to the identification of Chinese medicinal materials. Thus, DNA level identification is needed to differentiate between Guang-dilong and other species.

The loop-mediated isothermal amplification (LAMP) method is a new technique for nucleic acid amplification that has become one of the commonly used nucleic acid detection technologies with its unique advantages [8,9]. Four specific primers were designed for six different regions of the target gene. The LAMP system requires a strand-displacing DNA polymerase, Bst DNA polymerase, and the reaction can be completed at a constant temperature within 60 min without the complicated thermal denaturation process. Up to now, because of its efficiency, rapidity, and high specificity, LAMP technology has been widely used in the identification of various fields. According to previous reports, LAMP method has been widely used in the detection of various diseases in people [10,11]. Moreover, the diagnosis of plant pathology and food quality control can be well achieved by LAMP [12–15]. LAMP technique has also solved the problems of complicated procedures, expensive equipment, and time-consumption in the past. So far, researchers have used many biological techniques to identify herbal medicines, such as random amplified polymorphic DNA (RAPD), inter-simple sequence repeat (ISSR), and restriction fragment length polymorphism (RFLP), but all these methods are based on PCR, which is difficult to complete the detection in a short time. Instead of traditional PCR-based assays, LAMP protocol is a better choice for the rapid identification of crude drugs.

A LAMP technique based on *trn* K gene sequences was developed as a new method to identify *Curcuma longa* and *Curcuma aromatica*, which was the first report on the identification of herbal plants using LAMP-based assay [16]. In the last decade, LAMP method has been used to authenticate the various kinds of herbal medicines, such as *Catharanthus roseus, Hedyotis diffusa, Taraxacum formosanum*, and saffron etc. [17–21]. However, the application of LAMP technology in the identification of animal medicines has not been reported up to now. The cytochrome *c* oxidase I (COI) in the mitochondrial genome has been considered as a core DNA barcoding to diagnose and discover animal species [22–25]. Furthermore, COI gene has been used to discover new species, classify different species, and analyze the phylogeny for earthworms [26,27]. Therefore, the COI gene is a good target-gene choice for designing specific primers in LAMP.

In the present study, we designed primers using the COI gene of Guang-dilong as the target gene and successfully established a highly sensitive and specific LAMP reaction system. The LAMP method was used for the identification of commercial Guang-dilong for quality control. To the best of our knowledge, this is the first report of LAMP technology for the identification of Guang-dilong.

## Methods

### Samples

The samples of five earthworm species (*P. aspergillum, E. foetida, A. obscuritoporus, P. guillelmi*, and *M. magna*) were collected from different parts of China and were identified by Jing Sun at Nanjing Agricultural University, China, and Jibao Jiang at Shanghai Jiao Tong University, China (Table 2). All earthworm specimens were deposited at Guangdong Institute of Applied Biological Resources.

### Genomic DNA extraction

Approximately 30 mg of the dorsal muscle of the fresh earthworms was taken for DNA extraction using the TIANamp Genomic DNA Kit (Tiangen Biotech Co, Ltd., Beijing, China). We obtained the COI sequence of all samples and validated their origin of identification. The total DNA concentrations were determined by BioMate 3 (Thermo Fisher Scientific, U.S.A.). All DNA samples were stored at −20 °C.

### Primer design

Using universal primers (Table 1), the COI gene fragment of five species of earthworm (*P. aspergillum, E. foetida, A. obscuritoporus, P. guillelmi, M. magna*) was amplified by PCR. The sequence alignment of COI sequences was carried out by software MEGA 6.0. COI sequence of Guang-dilong and its adulterants were used to design LAMP primers. The Primer Explorer V5 software (http://primer explorer.jp) was used to design primers for LAMP.

**Table 1:** Primers used in the present study

Amplification method	Primers	Sequence(5′–3′)	Length
LAMP	FIP (F1c+F2)	CAACAGCCGCAGACCTTACTA-AACATAAGATTTTGACTTTTGCC	44
	BIP (B1c+B2)	GGACAGTTTACCCCCCTTTAGC-GCTAAATGTAGTGAGAAAATTGCA	46
	F3	CATAGCATTCCCACGTCTA	19
	B3	ACCTAAAATTGATGAGGCAC	20
Conventional PCR	HCO2198	TAAACTTCAGGGTGACCAAAAAATCA	26
	LCO1490	GGTCAACAAATCATAAAGATATTGG	25

### LAMP and PCR

The LAMP reaction occurred in a total volume of 25 μl that contained 2.5 μl of 10× Bst DNA polymerase reaction buffer, 1.6 mM of each of the inner primers (FIP and BIP), 0.2 mM of each outer primer (F3 and B3), 1.4 mM of dNTPs, 6 mM MgSO_4_,1 μl of *Bst* DNA polymerase (New England Biolabs), 1 μl DNA template. The mixture was first incubated at 95°C for 5 min, then placing reaction tube on ice for 1 min, followed by adding 1 μl of Bst DNA Polymerase, and the reaction was incubated at 65°C for 60 min and 80°C for 10 min to end the LAMP reaction. The reaction temperatures (58–67°C) and reaction times (10–60 min) were examined to obtain the best LAMP reaction conditions, and 67.5 ng/μl of the DNA concentrations was used in optimization and specificity tests of LAMP.

The conventional PCR method was developed based on the COI sequences of Guang-dilong. The PCR reaction was a 25-μl mixture containing 12.5 μl Taq PCR Mix, 1 μl DNA template, 1 μl of each primers (Table 1), reaction mix 7.5 μl, DNA polymerase 0.4 μl. The PCR cycle procedures were: 3 min at 95°C; then 35 cycles at 95°C for 30 s, 55°C for 30 s, and 72°C for 45 s; and final extension at 72°C for 10 min. The PCR amplification products were detected by 1% agarose gel electrophoresis.

### Detection of LAMP products

We used two different methods to detect LAMP products. The LAMP products were detected by naked eye observation of the color change after adding 1 μl of SYBR Green I dye (Invitrogen, U.S.A.), or the LAMP products can be analyzed by electrophoresis on 2% agarose gels.

## Results

### Sequence analysis and primer design

**Figure 2:**
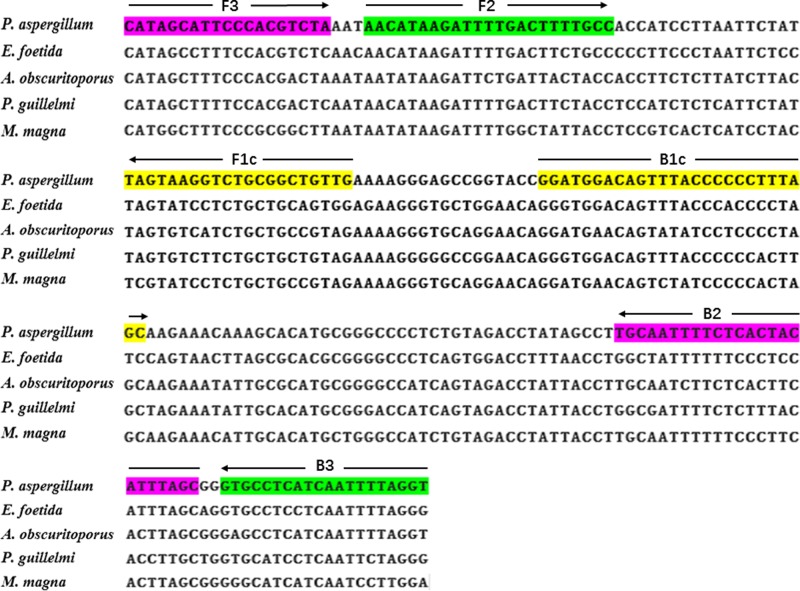
Sequence alignment of COI gene between *P. aspergillum* and its adulterants, and location of primer design for LAMP The sequences in six colored boxes represent the location for LAMP primers. The arrow symbols indicate the direction of 5′–3′ amplification in LAMP.

The variation of COI sequences between Guang-dilong and *E. foetida, A. obscuritoporus, P. guillelmi*, and *M. magna* was aligned as illustrated in Figure 2. Based on the interspecies variation of COI sequence, four primers were designed in six regions of COI sequence of Guang-dilong, including two outer primers (F3 and B3) and two inner primers (FIP and BIP) (Table 1).

### Optimization of LAMP conditions

**Figure 3:**
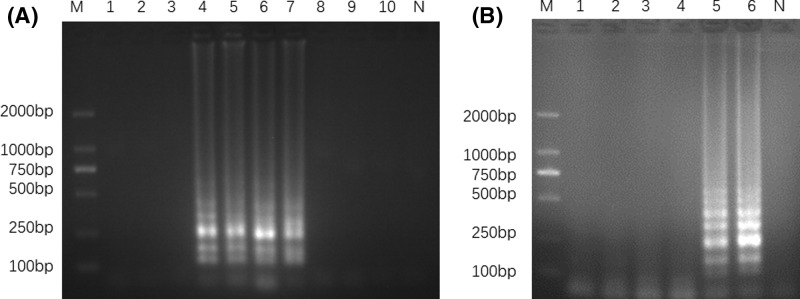
Optimization of temperature and time in LAMP Electrophoretic analysis of LAMP products at different temperatures and different times; (**A**) lane 1, 58°C; lane 2, 59°C; lane 3, 60°C; lane 4, 61°C; lane 5, 62°C; lane 6, 63°C; lane 7, 64°C; lane 8, 65°C; lane 9, 66°C; lane 10, 67°C. (**B**) Lane 1, 10 min; lane 2, 20 min; lane 3, 30 min; lane 4, 40 min; lane 5, 50 min; lane 6, 60 min. Lane M, 2000-bp ladder size marker; lane N, no template control (ddH_2_O).

To optimize the LAMP reaction conditions, different reaction temperatures (58–67°C) and different amplification times (10–60 min) were tested. The results showed that the reaction temperatures ranged from 61 to 64°C, no amplification was detected when the temperature was too low or too high (58–60 and 65–67°C), and the temperature of 63°C was found to be optimal in generating the best ladder-like bands (Figure 3A). The LAMP products was detected at 50 min, and the optimal amplification time for LAMP was determined to be 60 min (Figure 3B).

### Specificity and sensitivity of LAMP assays

**Figure 4:**
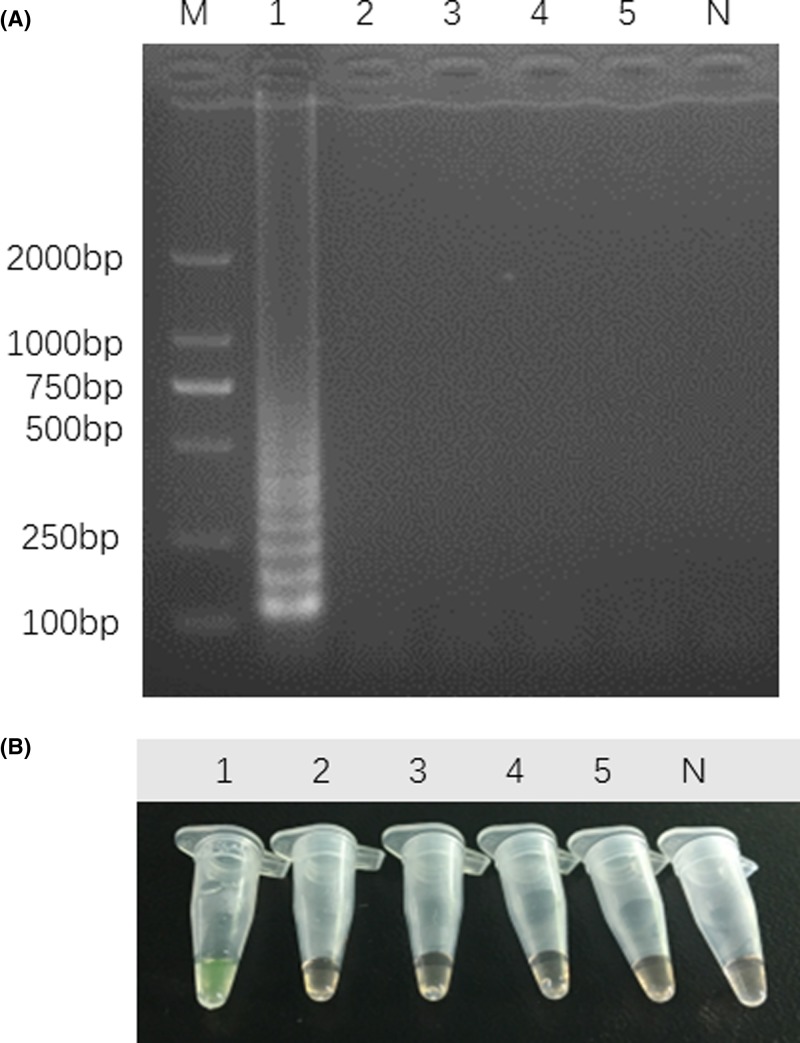
Specific identification for *P. aspergillum* using LAMP Electrophoresis (**A**) and visualization (**B**) for LAMP products detection. Lanes and tubes: 1, *P. aspergillum*; 2, *E. foetida*; 3, *A. obscuritoporus*; 4, *P. guillelmi*; 5, *M. magna*. Lane M, 2000-bp ladder size marker; lane N, no template control (ddH_2_O).

**Figure 5:**
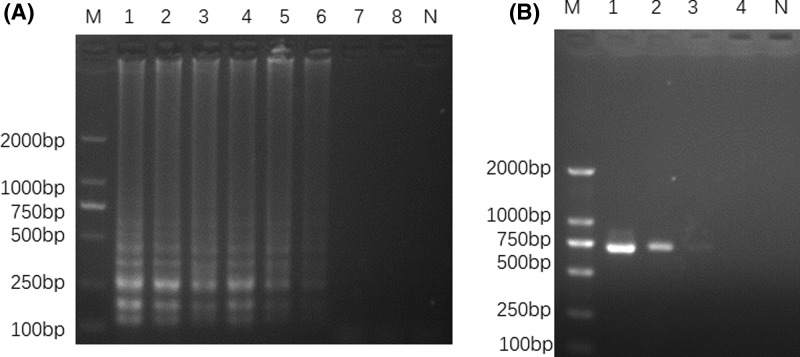
Determination of the sensitivity using LAMP and conventional PCR Amplification products of LAMP (**A**) and conventional PCR (**B**) were detected by gel electrophoresis; lane 1, 67.5 ng/μl; lane 2, 6.75 ng/μl; lane 3, 675 pg/μl; lane 4, 67.5 pg/μl; lane 5, 6.75 pg/μl; lane 6, 675 fg/μl; lane 7, 67.5 fg/μl; lane 8, 6.75 fg/μl. Lane M, 2000-bp ladder size marker; lane N, no template control (ddH_2_O).

We used Guang-dilong genomic DNA (positive sample) and genomic DNA from other four species (negative sample) as templates to detect the LAMP specificity. The result showed that ladder-like bands were observed in positive sample by electrophoresis, while no amplification occurred in negative samples (Figure 4A). Under natural light, we observed that positive tube changed from orange to green after adding SYBR Green I dye, whereas the negative and no template control tubes remained orange (Figure 4B). Therefore, the two detection methods were suitable for authentication of Guang-dilong from other species of earthworms in the present study. To test the sensitivity of LAMP, the Guang-dilong genomic DNA sample was serially diluted into different concentrations (67.5 ng/μl to 6.75 fg/μl). As depicted in Figure 5, the concentrations of DNA ranging from 67.5 ng/μl to 675 fg/μl were successfully amplified (Figure 5A). In contrast, conventional PCR method was used to amplify COI gene of Guang-dilong with conventional primers, but the detection limit for conventional PCR was only 675 pg/μl (Figure 5B), indicating that its sensitivity was 1000-times lower than that of the LAMP. Thus, the LAMP method was well used for identification of Guang-dilong with its high specificity and sensitivity.

### Application of LAMP method to the identification of commercial Guang-dilong

**Figure 6:**
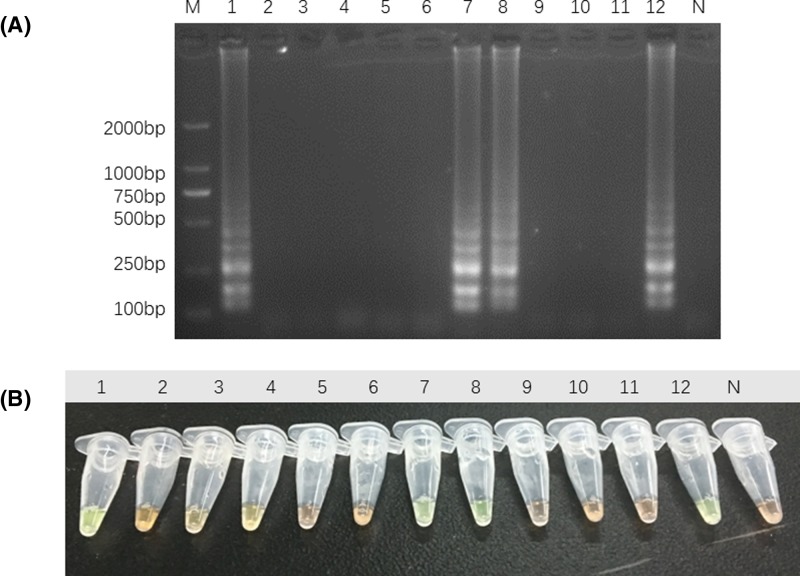
Identification of commercial Guang-dilong by LAMP Both electrophoretic assay (**A**) and visual inspection (**B**) were carried out for detection of LAMP product in different samples. Lanes and tubes: 1, simple GDL-06; 2, simple GDL-07; 3, simple GDL-08; 4, simple GDL-09; 5, simple GDL-10; 6, simple GDL-11; 7, simple GDL-12; 8, simple GDL-13; 9, simple GDL-14; 10, simple GDL-15; 11, simple GDL-16; 12, simple GDL-17. Lane M, 2000-bp ladder size marker; lane N, no template control (ddH_2_O).

Based on the establishment of LAMP evaluation system, we applied it as a practical method for the authentication of 12 samples from Taobao.com and herbal markets in China (Table 2). The genomic DNA of these samples were extracted as templates, following the steps in operation procedures of LAMP, the amplification products were detected by electrophoresis and visualization when the LAMP reactions were finished. The results, shown in Figure 6, revealed that the amplification products of 4 samples could be examined amongst 12 commercial samples of Guang-dilong, of which three were processed herbal products and one fresh herbal product (GDL-06, GDL-12, GDL-13, and GDL-17), respectively. The test result indicates that only one-third of commercial herbal medicines were identified as Guang-dilong.

**Table 2:** Information of samples in the study

Samples	Latin name	Characteristics	Source	Identifier
GDL-01	*P. aspergillum*	Fresh	Guangxi, China	Jing Sun
GDL-02	*E. foetida*	Fresh	Jiangsu, China	Jing Sun
GDL-03	*A. obscuritoporus*	Fresh	Guangdong, China	Jibao Jiang
GDL-04	*P. guillelmi*	Fresh	Shanghai, China	Jibao Jiang
GDL-05	*M. magna*	Fresh	Hainan, China	Jibao Jiang
GDL-06		Fresh	Yulin, Guangxi	
GDL-07		Fresh	Zhanjiang, Guangdong	
GDL-08		Fresh	Maoming, Guangdong	
GDL-09		Fresh	Zhaoqing, Guangdong	
GDL-10		Fresh	Yangjiang, Guangdong	
GDL-11		Fresh	Beihai, Guangxi	
GDL-12		Processed	Yulin herbal market, Guangxi	
GDL-13		Processed	Yulin herbal market, Guangxi	
GDL-14		Processed	Taobao.com	
GDL-15		Processed	Bozhou herbal market, Anhui	
GDL-16		Processed	Qingping herbal market, Guangzhou	
GDL-17		Processed	Qingping herbal market, Guangzhou	

## Discussion

Traditional methods of morphology, microscopy, and chemistry are reliable in the identification of fresh medicinal materials, but medicinal herbal products are usually sold in heavily processed forms. Confusion is often caused in the identification of processed herbal medicines when using these methods. DNA-based methods are extremely effective and can be applied for detection of various herbal medicines. DNA can be easily extracted from small amount of tissue of herbal products. As a new molecular biological technique, LAMP is obviously suitable for developing countries where there is lack of expensive testing equipment. LAMP method has changed our view on the complexity of identification procedures of herbal medicines. Furthermore, the safety of herbal medicines can be significantly improved when LAMP technique is applied to identification of herbal products, especially combined with morphological and other molecular methods. In the present study, we have successfully established LAMP assay, a rapid, highly sensitive, and specific tool for identification of Guang-dilong. The target gene requires highly conserved and sufficient length of sequence that was necessary for the design of specific primers. Previous studies have indicated that the nucleotide sequence of the mitochondrial COI gene, as DNA barcode for animals, could reflect the evolutionary relationship amongst the different species, which was especially adapted for the design of LAMP primers. We designed several groups of primers using Primer Explorer software based on the COI sequence gene of Guang-dilong. Through test screening, a set of species-specific primers with good reproducibility and high amplification efficiency was finally obtained, including FIP, BIP, F3, and B3 (Table 1). LAMP-based method usually completes the amplification within 60 min at 60–65°C. Specific conditions of reaction time and temperature were optimized for LAMP reaction in the present study. The result showed that the amplification products can be inspected after 50 min and the reaction temperature should be controlled ranging from 61 to 64°C. There are several dyes commonly used to detect the LAMP product, such as SYBR Green I, hydroxy naphthol blue (HNB), and calcein. SYBR Green I was added when the LAMP amplification was accomplished, while HNB and calcein can be added to the reaction system before the start of the reaction. We selected SYBR Green I for LAMP product detection because of its higher sensitivity [28]. Additionally, same with conventional PCR, the reaction products can also be detected by agarose gel electrophoresis. There were no false-positive or false-negative results for sample tests in our study. To decrease the probability of contamination, we should strictly follow the standard operating procedures. The experimental environment should always be exposed to UV light to ensure that it is not contaminated by amplification products. The reagents, the equipments, and pure water must be autoclaved before using. Besides, the reagent preparation area and the detection area must be strictly separated. If the false-positive results occur, we must stop the detection immediately, change the experimental environment, and use new reagents the next time. Therefore, we must carry out the sample testing in accordance with standard operating procedures of LAMP.

It is known that PCR-based techniques require sophisticated and expensive equipment to meet thermal cycling conditions and the whole operation process needs approximately 2–3 h, which is time-consuming for the field-test. Moreover, PCR techniques require large amounts of DNA template to perform the reaction. In contrast, LAMP-based assay is quite simple, only a water bath or heating block is enough, the reaction proceeds under the conditions of constant temperature (60–65°C), because LAMP-based detection requires Bst DNA polymerase that plays an important role with its strong strand-displacement activity [29]. The detection limit of LAMP assay was 1000-fold higher than conventional PCR in our study, that is a good news for poorly preserved or fragmentary herbal medicines because only small amounts of DNA are needed for identification. The LAMP operation process only takes approximately 1.5 h, which is very useful for the rapid identification of herbal medicines *in situ*. Due to the increase in the price of Guang-dilong in recent years, as well as reduction in its resources, high profits make the adulteration of commercial medicines a common phenomenon in Chinese herbal markets. To address these issues, new rapid methods should be established for the identification of Guang-dilong to protect human health. Luckily, this molecular biological technique was successfully developed for the identification of Guang-dilong in herbal markets. In our work, different sources of commercial samples were tested by LAMP technique, the results showed that two-thirds of samples were substituted for Guang-dilong, indicating its adulterants account for a large proportion of Guang-dilong products in Chinese herbal markets.

## Abbreviations

COI: cytochrome *c* oxidase I
HNB: hydroxy naphthol blue
LAMP: loop-mediated isothermal amplification
